# Toward a thermodynamic stability order of the phosphorus allotropes

**DOI:** 10.1039/d5ra06696d

**Published:** 2025-11-06

**Authors:** Laura Bonometti, Giuseppe Sansone, Marcos Rivera-Almazo, Denis Usvyat, Antti J. Karttunen, Lorenzo Maschio

**Affiliations:** a Dipartimento di Chimica, Università di Torino Via P. Giuria 5 10125 Torino Italy; b Institut für Chemie, Humboldt-Universität zu Berlin Brook-Taylor-Str. 2 D-12489 Berlin Germany; c Department of Chemistry and Materials Science, Aalto University Kemistintie 1 02150 Espoo Finland antti.karttunen@aalto.fi; d Dipartimento di Chimica, NIS Centre, Università di Torino Via P. Giuria 5 10125 Torino Italy lorenzo.maschio@unito.it

## Abstract

In this work, we investigate through quantum–mechanical calculations the relative stability of white-γ, white-β, fibrous red, violet, and orthorhombic black phosphorus allotropes, a longstanding yet challenging problem. We performed DFT-D3 calculations with the CRYSTAL code as well as periodic local second-order Møller–Plesset perturbation theory (p-LMP2) calculations. DFT and Spin-Component Scaled p-LMP2 place violet phosphorus as the thermodynamically most stable allotrope both at 0 K and at 298 K, yet within a tiny margin from the black phosphorus. Pure p-LMP2 suggests that black phosphorus is the most stable, although its accuracy may be affected by the narrow band gap in this material.

## Abstract 2

In this work, we investigate through quantum–mechanical calculations the relative stability of white-γ, white-β, fibrous red, violet, and orthorhombic black phosphorus allotropes, a longstanding yet challenging problem.

## Graphical abstract

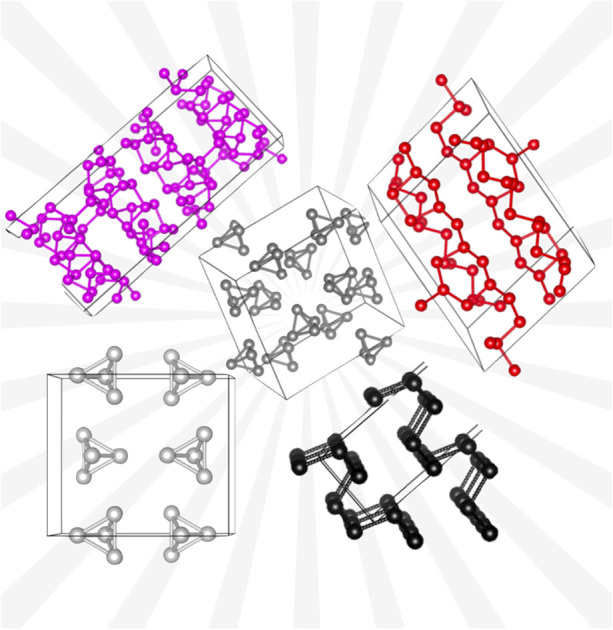

## 1 Introduction

The rich allotropy of phosphorus makes it an exciting element from the point of view of fundamental structural chemistry and structure–property correlations. The bulk structural modifications of phosphorus are usually described based on a color scheme that divides the allotropes to the white, red and black phosphorus classes.^1–4^ During the past 15 years, various nanostructured and low-dimensional allotropes of phosphorus have also been characterized.^5–9^ At the ambient pressure, there are both amorphous and crystalline forms of phosphorus, and we start by briefly summarizing most known and well-characterized bulk crystalline allotropes.

The white phosphorus modifications are based on P_4_ tetrahedra, and there are two different crystalline forms, namely β and γ.^10–15^

The red phosphorus class exhibits the greatest structural diversity^2^ and only two crystalline allotropes are known: fibrous red (red-IV) and violet (Hittorf's, red-V) phosphorus.^16–23^ They are made of tubular P units that form complex double layers by connecting perpendicularly in violet phosphorus and in parallel in fibrous red phosphorus. The layers are then stacked in the three-dimensional crystal structure, stabilized by weak van der Waals forces.

Several modifications also exist in the black phosphorus class. At ambient temperature and high pressures (20 °C; 12 kbar), crystalline orthorhombic black phosphorus can be obtained, which shows a layered structure composed of puckered six-membered P rings. The layers are kept together also by van der Waals interactions.^3,4,11,24^

Bridgman's discovery of the orthorhombic black phosphorus with layered structure encouraged further studies to determine the structural and thermodynamical relations of the phosphorus allotropes.^11^ Orthorhombic black phosphorus^25,26^ is typically described as the thermodynamically most stable form of the element at NTP conditions.^3,4^ However, re-tracing the main historic studies on the topic, Aykol *et al.*^27^ have suggested that there is little direct evidence to support this claim.^11,19,24,28–35^ Nilges *et al.*^1^ have discussed the difficulties in obtaining hard thermodynamic evidence on the stability order of bulk phosphorus allotropes from temperature-dependent vapor-pressure measurements.

The structural variety of phosphorus allotropes and the experimental difficulties in pinpointing the thermodynamic stability order have led in several systematic computational investigations within this field with density functional theory (DFT).^27,36–39^ New phosphorus modifications are also being proposed and predicted theoretically, confirming the lively interest in this element.^40^ Furthermore, machine-learning interatomic potentials based on DFT plus many-body dispersion approach have enabled the investigation of both crystalline and amorphous phosphorus modifications in size scale that was inaccessible few years ago.^41–44^

From a computational point of view, one of the most challenging aspects of the thermodynamical comparison of various phosphorus allotropes is the coexistence of covalent bonding and weak dispersive interactions. Standard DFT methods do not describe the weak van der Waals interactions in phosphorus allotropes properly and dispersion-corrected DFT methods are needed.^45–50^ While several dispersion-corrected DFT approaches are available, they need to be benchmarked carefully to ensure that all classes of phosphorus allotropes are described with equal accuracy.

**Fig. 1:**
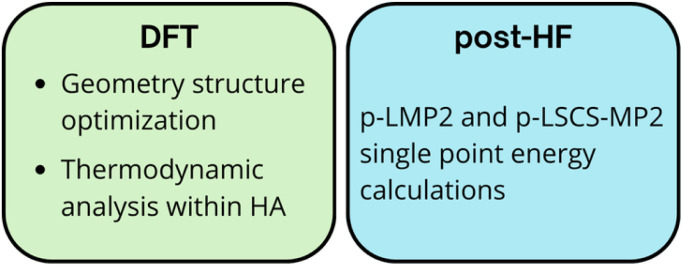
Schematic workflow of this study.

**Fig. 2:**
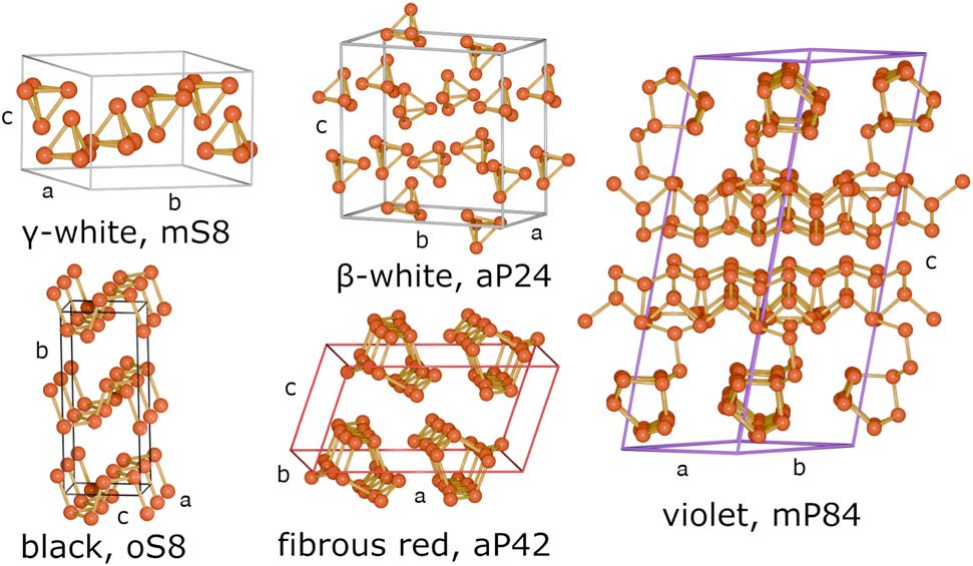
Structures of the phosphorus allotropes listed in Table 1.

In this work (Fig. 1), we investigate the thermodynamics of bulk crystalline phosphorus allotropes that exist at ambient pressure, deliberately excluding the high-pressure phases, such as those of black phosphorus,^36,51^ which lie beyond the scope of this study. The studied phosphorus allotropes are listed in Table 1 and their crystal structures are illustrated in Fig. 2. Dispersion-corrected DFT methods are used to fully optimize the structures and determine the energetic and thermodynamic stability of the phosphorus allotropes, together with other thermodynamic quantities such as the entropy and heat capacity. For an *ab initio* description of dispersion we also utilize periodic local second-order Møller–Plesset perturbation theory (p-LMP2).

**Table 1:** List of phosphorus allotropes studied in this work, together with their Pearson symbol, space group, and inorganic crystal structure database reference number^52^

Allotrope names	Pearson	Space group	ICSD no.	Ref.
White-γ	*mS*8	*C*2/*m*	154 318	14
White-β	*aP*24	P1̄	68 326	12
Fibrous, red IV	*aP*42	P1̄	391 323	21
Violet, red V	*mP*84	*P*2/*c*	29 273	22
Orthorhombic, black	*oS*8	*Cmca*	23 836	53

We believe that a coherent dataset of structures, energies and thermodynamic functions provides a robust benchmarking reference, which complements previous theoretical efforts to determine the most stable phosphorus allotrope.

## 2 Computational details

All DFT calculations were performed with the CRYSTAL code, which adopts atom-centered Gaussian-type functions for expanding crystal orbitals.^54^ PBE0 hybrid exchange-correlation functional^55^ was chosen while van der Waals dispersion interactions were accounted using Grimme's empirical DFT-D3 approach with zero-damping (ZD).^56^ Doubly polarized triple-valence quality basis set (TZVPP), derived from the molecular Karlsruhe basis set,^57,58^ was used in all the calculations, DFT and p-MP2. The SI includes detailed information on the performance of the selected level of theory with respect to the experimental crystal structures.

For sampling the reciprocal space, Monkhorst–Pack type *k*-meshes were used: 4 × 4 × 6 for *mS*8, 6 × 2 × 2 for *aP*24, 3 × 3 × 5 for *aP*42, 4 × 4 × 1 for *mP*84 and 6 × 6 × 6 for *oS*8.^59^ Both nuclear coordinates and lattice parameters were relaxed for the geometry optimization of all studied structures, conserving the space group symmetry. Default DFT integration grids and optimization criteria of CRYSTAL23 were applied. The five thresholds *T*_i_, which control the truncation criteria of the Coulomb and exchange infinite lattice series, have been set to 8 (*T*_1_–*T*_4_) and 16 (*T*_5_). To properly describe the thermodynamic properties of the allotropes, we sampled phonon modes outside the *Γ*-point by using phonon supercells as implemented in the CRYSTAL code.^54,60^ The applied phonon supercells and the *k*-meshes used in the phonon calculations are reported in SI.

Then, we considered the optimized geometries at PBE0-D3(ZD)/TZVPP level of all the studied systems for single-point calculations with the periodic local second-order Møller–Plesset perturbation theory (p-LMP2) and its spin-component-scaled variant^61^ (p-SCS-LMP2).^62–65^ As the HF reference for p-LMP2, we used the AO-based periodic HF of the CRYSTAL code.^54^

In the p-LMP2 method the occupied manifold is represented by local Wannier functions (WFs),^66,67^ allowing one to exploit the short-range nature of electron correlation and to achieve a nearly linear scaling with the number of atoms per cell. As virtual functions p-LMP2 employs the so-called orbital specific virtuals (OSVs),^68,69^ which provide an efficient and compact representation of the pair-specific virtual space. The two-electron integrals in p-LMP2 are approximated *via* local density fitting.^70^

## 3 Results and discussion

### 3.1 Structure optimizations

Table 2 reports the essential structural information for the crystal structures optimized at the PBE0-D3(ZD)/TZVPP level of theory while more comprehensive details are provided in the SI of one of our recent studies on the phonon frequency analysis of the IR and Raman spectra of these phosphorus allotropes.^71^ Our choice of this level of theory is guided by a previous benchmark performed on black phosphorus.^72^ where different DFT functionals and strategies were evaluated to account for London dispersion forces (including the Tkatchenko–Scheffler (TS) method^73^ and many-body dispersion model (MBD)^74^) were assessed, and this combination emerged as the most accurate. For consistency with earlier studies and reliability demonstrated by that benchmark, we retained this computational approach. The optimized lattice parameters agree well with the experimental data for allotropes studied. The largest deviation in lattice parameters is 1.3% for the *b* parameter of the *oS*8 allotrope (corresponding to the stacking direction of the layers in black-P). The good performance of PBE0-D3(ZD)/TZVPP in predicting the minimum energy geometries indicated that this functional could also be used further for investigation of the thermodynamic properties.

**Table 2:** Lattice parameters of the studied phosphorus allotropes optimized at PBE0-D3(ZD)/TZVPP level of theory. *a*, *b* and *c* are given in Å, *α*, *β* and *γ* in degrees, and volumes *V* in Å^3^. The percentage values in parentheses show the differences compared to experimental values. 90° angles are omitted

Param.	*mS*8	*aP*24	*aP*42	*mP*84	*oS*8
*a*	9.25 (+0.9%)	5.48 (0.0%)	12.27 (+0.6%)	9.22 (+0.1%)	3.30 (−0.1%)
*b*	8.25 (−1.1%)	10.86 (+0.6%)	13.00 (+0.1%)	9.13 (−0.2%)	10.61 (+1.3%)
*c*	5.45 (+0.4%)	11.08 (+1.1%)	7.11 (+0.5%)	22.65 (+0.2%)	4.42 (+0.9%)
*α*	—	93.9 (−0.4%)	116.9 (−0.1%)	—	—
*β*	90.5 (+0.2%)	99.7 (0.0%)	106.4 (+0.1%)	106.0(−0.1%)	—
*γ*	—	101.2 (+0.5%)	97.9 (0.0%)	—	—
*V*	415.8(+0.1%)	634.4 (+1.7%)	922.8 (+1.3%)	1832.9 (+0.2%)	154.8 (+1.9%)

### 3.2 Thermodynamic calculations

For most solids, thermodynamic properties obtained within the harmonic approximation are known to be rather accurate for temperatures much smaller than the melting temperature or any phase transition temperature.^75^ A more accurate description of the thermodynamic behavior can be achieved with the quasiharmonic approximation (QHA), as the latter can be valid up to temperatures between the Debye temperature and the melting temperature for thermodynamically stable crystals.^76,77^ Despite its significantly lower computational cost compared to molecular dynamics at a similar level of theory, it retains reasonable accuracy in capturing thermal expansion.^78^ However, when soft vibrational modes are present, QHA still may fail even below the Debye temperature.^79^ Unfortunately, the *Γ*-point vibrational modes of the phosphorus allotropes show several rather soft modes, originating from the weak van der Waals binding between the molecules in *mS*8 and *aP*24, fibers in *aP*42 or *mP*84 or layers in *oS*8, as observed in a previous work of our group where QHA calculations were performed on black phosphorus, highlighting its well-known strong anisotropy. This suggests that QHA may not bring a significant leap in accuracy over the standard harmonic approximation. As the computational cost of QHA calculations for some of the studied systems is rather high, we restrict our calculations to the harmonic approximation. We note therefore that the thermodynamic data reported here, especially for the higher temperatures, should be considered within the limitations of this approximation.

**Fig. 3:**
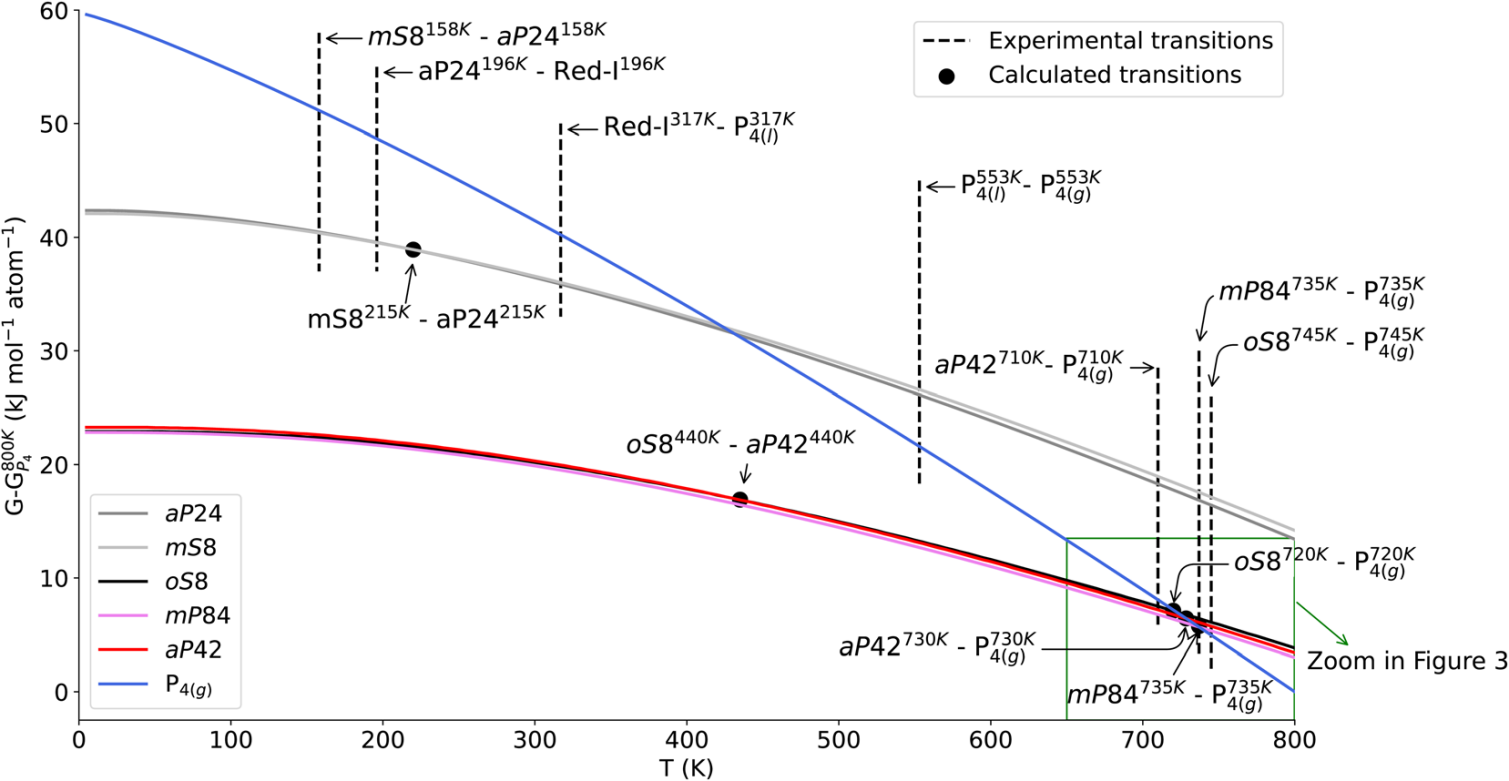
Gibbs free energy curves of the studied phosphorus allotropes and the gas-phase P_4_ molecule. The zero level is the Gibbs free energy of P_4_ at 800 K. The symbols highlight the calculated crossovers between the Gibbs free energies of the allotropes. Some experimental phase transitions are reported as vertical lines, according to the ref. 1 for *mP*84 and *oS*8 allotropes transitions to P_4_(g); to ref. 12 for experimental transition from γ to β white modification; to ref. 34 for the transition from *aP*42 to P_4_(g); to ref. 3 for transition from red-I to P_4_(l) and to ref. 4 for all the remaining transitions. The inset highlighted by the green rectangle is shown in high detail in Fig. 4. Red-I refers to the amorphous red phosphorus.

The temperature-dependent Gibbs free energy curves at ambient pressure for the solid-state phosphorus allotropes and for the isolated (gas-phase) P_4_ molecule are shown in Fig. 3, where also experimental phase transitions are reported to be compared with our results. Some of the experimental results could not be simulated as they involve amorphous phosphorus allotropes such as as amorphous red phosphorus (red-I) or α-white modification.

First we note that, as concerns the white modifications *mS*8 and *aP*24, the calculated results agree reasonably well with the experiment. The computed values indicate that these allotropes are substantially less thermodynamically stable than the black or red/violet phosphorus, which is not surprising as white phosphorus modifications are weakly bound molecular crystals. Experimentally this manifests itself in relatively low temperatures of the phase transition from the *aP*24 to the amorphous red-I phase and later from red-I to the liquid and gas phases. Since the amorphous phases are not directly accessible computationally, the calculation considers a direct transition from *aP*24 to the gas phase. The predicted temperature of such a transition is slightly underestimated compared to the experimental temperatures, which may suggest that the PBE0-D3 underestimates the dispersive interactions in white phosphorus (see also discussion below on the p-LMP2 results) or that the harmonic approximations fails for the soft intermolecular modes (or both).

According to our calculations the two allotropes of white phosphorus are energetically very close to each other at all temperatures, within sub-kJ mol^−1^ per atom, see Table 3. At high temperatures the *aP*24 modification is slightly more stable than mS8, at low temperatures the stability order reverts. This trend agrees with the experimental phase transition from *mS*8 to *aP*24 by heating. Notably, the calculated phase-transition temperatures align fairly well with experiment (see Fig. 3), which is rather remarkable given the underlying harmonic approximation and the relative crudeness of the D3 dispersion model.

**Table 3:** The relative electronic energies (Δ*E*) and Gibbs free energies (Δ*G*) at 0, 298, and 500 K (in kJ mol^−1^ per atom). The *oS*8 allotrope (black-P) is used as the reference state

Allotrope	Δ*E*	Δ*G*_0_	Δ*G*_298_	Δ*G*_500_
*mS*8	20.0	19.2	16.4	14.0
*aP*24	20.3	19.4	16.3	13.6
*aP*42	0.4	0.4	0.1	−0.1
*mP*84	−0.1	−0.1	−0.3	−0.5
*oS*8	0.0	0.0	0.0	0.0

**Fig. 4:**
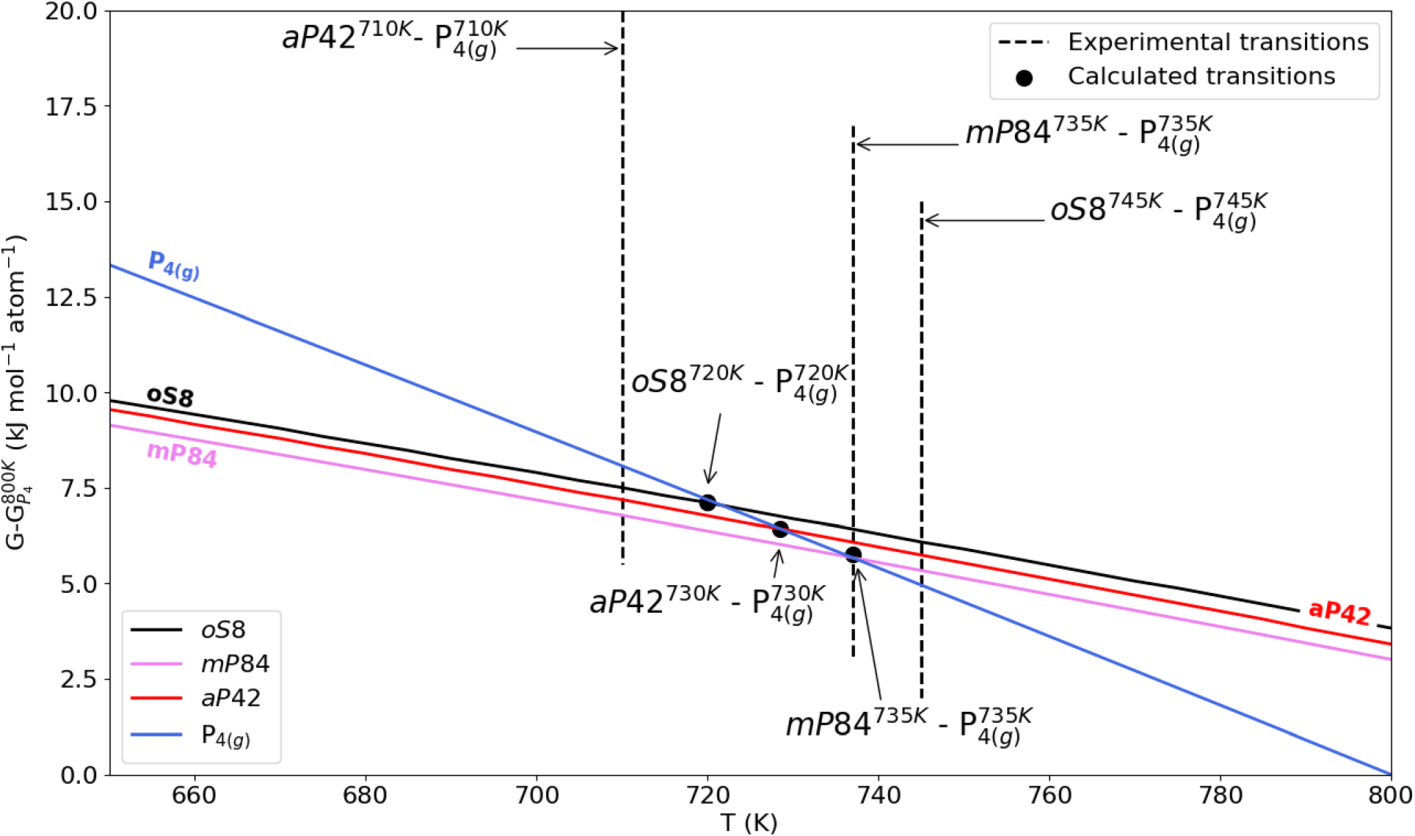
Zoomed-in portion of Gibbs free energy curves in between 650 and 800 K of *os*8, *aP*42 and *mP*84 allotropes and the gas-phase P_4_ molecule. The zero level is the Gibbs free energy of P_4_ at 800 K. The symbols highlight the calculated crossovers between the Gibbs free energies of the allotropes. Experimental phase transitions are reported as vertical lines, according to the ref. 1 for *mP*84 and *oS*8 allotropes transitions to P_4_(g) and to ref. 34 for transition from from *aP*42 to P_4_(g).

Next we focus on the other three allotropes and the temperature region of the phase transitions to the gas phase, which is shown in detail see Fig. 4. Experimentally the temperatures of such phase transitions lie in a very small window, with the temperature for the black phosphorus' transition being the highest. The calculations do not exactly reproduce this order, most likely due to the harmonic approximation, possibly in combination with the approximations within DFT and the dispersion model employed. Indeed, given the presence of soft and likely anharmonic inter-layer or inter-fiber modes, it is not surprising that the harmonic approximation does not correctly resolve the tiny differences in the Gibbs free energies at high temperatures. Nevertheless, and more importantly, our calculations reproduce the proximity of the phase transition temperatures and furthermore predict the temperature range for these transitions very accurately. In Fig. 3 and Table 4 all relevant experimental and calculated phase transitions temperatures are illustrated and listed.

**Table 4:** Experimental and calculated phase transition temperatures (K) of P allotropes (see Fig. 3 and 4)

Phase transition	Exp. *T*	Calc. *T*
*mS*8 – *aP*24	158 [ref. 12]	215
*aP*24 – Red-I	196 [ref. 4]	—
Red-I – P_4(l)_	317 [ref. 3]	—
*oS*8 – *aP*42	—	440
P_4(l)_ – P_4(g)_	553 [ref. 4]	—
*aP*42 – P_4(g)_	710 [ref. 34]	730
*mP*84 – P_4(g)_	735 [ref. 1]	735
*oS*8 – P_4(g)_	745 [ref. 1]	720

**Fig. 5:**
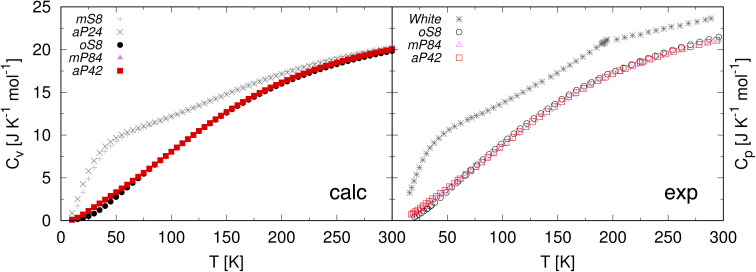
Left: heat capacities at constant volume predicted in this work. Right: experimental heat capacities at constant pressure.^19^ Three different forms of white phosphorus, white-β, white-γ, and amorphous white are present in the temperature range, but they are not distinguished in the experimental data.

For a further insight in the accuracy of our calculated Gibbs free energies, we compared our results with the experimental heat capacity and entropy data. Stephenson *et al.*^19^ reported the constant pressure heat capacities of phosphorus allotropes from about 15 up to 300 K, together with entropies at low temperatures and at 298 K. Other data are provided by ref. 80 and 81. Paukov *et al.*^82^ published data for the *oS*8 allotrope, but according to Stephenson *et al.* their data differ only about 1%.^19^ Comparisons of the entropies and heat capacities are reported in Tables 5 and 6 respectively. The entropy data show a good overall agreement between the calculations and the experiments. The calculated trends of the heat capacities also agree well with the experimental ones, see Fig. 5. In particular, the low-temperature behavior of the *mS*8 and *aP*24 allotropes is well reproduced. Above 158 K, *mS*8 converts to *aP*24, which in turn transforms to amorphous white phosphorus at about 196 K.^12^ Right in this region, from about 150 K, the slopes of the experimental curves change in a way that indicate the upcoming phase transition.

**Table 5:** Calculated and experimental entropies of the studied phosphorus allotropes at 298 K (in J K^−1^ mol^−1^ units). White refers to amorphous white phosphorus

Allotrope	*S*_calc_	*S*_exp_^19^	*S*_exp_^80^	*S*_exp_^81^
White	—	41.00	41.08	41.09
*mS*8	32.2	—	—	—
*aP*24	33.6	—	—	—
*aP*42	21.3	23.17	23.20	—
*mP*84	21.3	22.84	22.85	22.80
*oS*8	20.4	22.59	22.59	—
*P*_4_	290.2	279.07	279.99	279.98

**Table 6:** Calculated heat capacity at constant volume (*C*_v_) and experimental heat capacities at constant pressure (*C*_p_) for the studied phosphorus allotropes (at 298 K in J K^−1^ mol^−1^ units). White refers to amorphous white phosphorus

Allotrope	*C*_v_	*C*_p_^19^	*C*_p_^80^	*C*_p_^81^
White	—	23.82	23.83	23.84
*mS*8	20.3	—	—	—
*aP*24	20.2	—	—	—
*aP*42	19.0	21.26	21.26	—
*mP*84	20.0	21.19	21.19	21.21
*oS*8	19.8	21.55	21.55	—
*P*_4_	57.7	—	67.16	67.15

Finally, we look at the low-temperature regime for the black and red/violet phosphorus allotropes. As the temperatures drops, the *oS*8 is predicted to become more stable than *aP*42. However it still remains slightly above *mP*84 at any temperature (see Fig. 3 and Table 3), with a minute energy difference of −0.1 kJ mol^−1^ per atom in favor of the latter at *T* = 0. This result is in line with the predictions of other theoretical investigations. Generally the sign of the relative stability between *oS*8 and *mP*84 may vary depending on the functional employed.^27,38^ However calculations using high-level dispersion models, such as the random phase approximation (RPA)^27^ or HSE06+MBD (many-body-dispersion correction)^44^ both predict *mP*84 to be more energetically stable, albeit by a tiny amount.

### 3.3 Post-HF calculations

In order to shed further light on the puzzle concerning the relative stability between black and violet phosphorus we employed a method outside the DFT domain with an *ab initio* treatment of dispersion interactions: periodic Local second-order Møller–Plesset perturbation theory. We performed single point p-PMP2 calculations on PBE0-D3 optimized structures. The p-LMP2 and p-SCS-LMP2 relative energies for the five phosphorus polymorphs are compiled in Table 7.

**Table 7:** p-LMP2 and p-SCS-LMP2 relative stabilities (Δ*E*) in kJ mol^−1^ per atom for the different phosphorus allotropes. The *oS*8 allotrope (black-P) is used as the reference state

Allotrope	Δ*E*^p-LMP2^	Δ*E*^p-SCS-LMP2^
*mS*8	12.74	9.78
*aP*24	13.81	10.38
*aP*42	3.61	1.99
*mP*84	1.49	−0.19
*oS*8	0.00	0.00

Interestingly, p-LMP2 predicts the black phosphorus to be the most stable allotrope. However the p-LMP2 result should be interpreted with caution as MP2 is known to overestimate dispersion in polarizable systems in general. Furthermore, MP2 is not able to capture Coulomb screening, which effectively weakens the long-range part of the dispersive interactions in bulk materials. Such screening effects are strong in narrow gap semiconductors, which is an additional source for overestimation of the interaction energy by MP2. Black phosphorus is a particular example, where pure p-LMP2 is known to significantly overestimate the exfoliation energy.^49^ We note that finite-temperature effects on electronic band gaps arise from electron–phonon coupling and can modulate correlation energies. Explicit inclusion of electron–phonon coupling for bulk black phosphorus is, however, beyond the computational scope of the present study. Nonetheless, experimental data indicate a temperature dependence of the band gap of about 
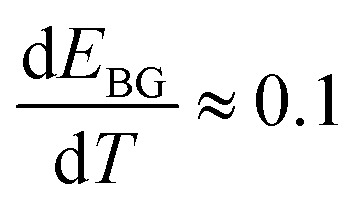
 meV T^−1^ in between 100 and 300 K^83^ and such a small absolute variation is unlikely to qualitatively affect our conclusions regarding MP2 overbinding. The violet phosphorus still being a narrow gap semiconductor, has a wider gap than a black phosphorus (*e.g.* the PBE0 gaps in our calculations: 0.90 eV for oS8 and 2.77 eV for mP84). Therefore p-LMP2 is expected to be less inaccurate for the violet phosphorus than for the black one, resulting in an artificial relative overstabilization of the latter.

The SCS-MP2 technique was introduced^84^ to repair the MP2 overbinding in highly polarizable systems. Therefore, p-SCS-LMP2 results for our systems are expected to be more balanced. And indeed the relative stability with p-SCS-LMP2 is again reversed, making the violet-phosphorus the most stable allotrope (yet again by a just −0.2 kJ mol^−1^ per atom). That is particularly interesting, as p-SCS-MP2 does not capture the Coulomb screening effects either^50^ and is expected to still be somewhat biased towards black phosphorus, although not as much as p-LMP2. And yet, it already favors the violet phosphorus, in line with the other high-level methodologies like RPA^27^ or HSE06 + MBD^44^ or HSE06 − MBD,^44^ which do include the screening effects.

As concerns other studied allotropes, the p-SCS-LMP2 results qualitatively follow the PBE0-D3 order of relatives stabilities. There is however a quantitative disagreement for the stability of the white phosphorus modifications: p-SCS-LMP2 predicts the white phosphorus to be energetically much closer to the black phosphorus than PBE0-D3. This result may be another indication that PBE0-D3 noticeably underestimates binding in white phosphorus, as suggested by the discrepancy in the predicted temperature of the phase transition to the gas phase (see above). Other theoretical estimates of the energy difference between the white and black phosphorus are about 15 kJ mol^−1^ per atom,^1,27,38^ which is somewhat in between the p-SCS-LMP2 and PBE0-D3 predictions.

## 4 Conclusion

In this work we studied the relative thermodynamical stability between five crystalline phosphorus modifications, namely *mS*8, *aP*24, *oS*8, *mP*84 and *aP*42 at PBE0-D3(ZD)/TZVPP levels of theory and SCS-LMP2(OSV)/TZVPP levels of theory, delivering systematic benchmarks and thermodynamic data for five crystalline allotropes within a single, consistent framework.

DFT and p-SCS-LMP2 at 0 K predict that the violet allotrope is energetically more stable than black phosphorus, but only minutely: by just 0.1–0.2 kJ mol^−1^ per atom. The p-LMP2 prediction reverts this order, but most likely due to the gross overestimation of dispersion in black phosphorus due to its very narrow gap.

Even if normalizing the energy difference between violet and black phosphorus by a characteristic unit like P_4_ or a unit cell of black phosphorus (eight atoms), it still remains clearly below the error margin of the methods employed here or in the other studies. Therefore, with confidence one can only state that these two allotropes are nearly isoenergetic. However, given the agreement between very different and relatively high-level theoretical approaches, one can cautiously speculate that it is the violet phosphorus being the most stable allotrope at low temperatures. For the other studied modifications the predicted order – fibrous red → γ-white → β-white—agrees with experimental data and other calculations.

At higher temperatures, PBE0-D3 with harmonic approximation reasonably accurately captures the temperature of the phase transition between the γ-white and β-white phosphorus, but somewhat underestimates their hypothetical sublimation temperatures. This suggests that PBE0-D3 may noticeably underestimate the formation energy of the molecular crystals of white phosphorus. For the black and red/violet phosphorus the predictions of their sublimation temperatures, which are very close to each other, are quite accurate. However, the order of the temperatures is not reproduced probably due to the deficiencies of the harmonic approximation.

## Conflicts of interest

There are no conflicts to declare.

## Supplementary Material

RA-015-D5RA06696D-s001

## Data Availability

The data supporting this article have been included as part of the supplementary information (SI). Supplementary information is available. See DOI: https://doi.org/10.1039/d5ra06696d.
