# Supplementary material for: Toward a thermodynamic stability order of the phosphorus allotropes
Source: RSC Adv. 2025 Nov 6;15(50):43028–35. doi: 10.1039/d5ra06696d (PMC12590358; doi:10.1039/d5ra06696d)
Supplement: RA-015-D5RA06696D-s001 [file RA-015-D5RA06696D-s001.pdf]

Supplementary Information (SI) for RSC Advances.
This journal is © The Royal Society of Chemistry 2025

Supporting information for: Toward a thermodynamic stability order of the phosphorus allotropes

Laura Bonometti, Giuseppe Sansone, and Marcos Rivera-Almazo
Dipartimento di Chimica, Università di Torino,
Via P. Giuria 5, 10125 Torino, Italy

Denis Usvyat
Institut für Chemie, Humboldt-Universität zu Berlin,
Brook-Taylor-Str. 2, D-12489 Berlin, Germany

Antti J. Karttunen
Department of Chemistry and Materials Science,
Aalto University, Kemistintie 1, 02150 Espoo, Finland[*]

Lorenzo Maschio
Dipartimento di Chimica, NIS centre, Università di Torino,
Via P. Giuria 5, 10125 Torino, Italy[†]

(Dated: October 4, 2025)

[*] antti.karttunen@aalto.fi
[†] lorenzo.maschio@unito.it

I. ADDITIONAL COMPUTATIONAL DETAILS

Table I reports the phonon supercells and the corresponding k-meshes used for the calculation of thermodynamic properties.

Table I. Phonon supercells and the corresponding k-meshes used for the calculation of thermodynamic properties. The supercells were built from the primitive cells, except for the allotrope $oS8$ where the crystallographic cell was used.

Parameter	$mS8$	$aP24$	$aP42$	$mP84$	$oS8^b$
$\mathbf{k}$-mesh	4x4x6	6x2x2	2x2x6	4x4x1	6x6x6
SC expansion matrix	$\begin{pmatrix}2&0&0\\0&2&0\\0&0&2\end{pmatrix}$	$\begin{pmatrix}2&0&0\\0&1&0\\0&0&1\end{pmatrix}$	$\begin{pmatrix}1&0&0\\0&1&0\\0&0&2\end{pmatrix}$	$\begin{pmatrix}1&0&0\\0&1&0\\0&0&1\end{pmatrix}$	$\begin{pmatrix}3&-3&0\\1&1&0\\0&0&2\end{pmatrix}$
a^{SC},b^{SC},c^{SC} (Å)	12.40,12.40,10.86	10.96,10.79,10.96	12.20,12.99,14.16	9.21,9.15,22.60	9.93,10.47,8.76
$\mathbf{k}$-mesh^{SC}	3x3x3	3x3x3	3x3x3	3x3x1	3x3x3

II. EFFECT OF THE DISPERSION CORRECTION

We benchmarked the performance of plain DFT-PBE0, DFT-PBE0-D3 with zero-damping (ZD) and DFT-PBE0-D3(ZD) with three-body correction (ABC).[1, 2] The results of the benchmarks are reported in Table II.

[1] S. Grimme, J. Antony, S. Ehrlich, and H. Krieg, A consistent and accurate ab initio parametrization of density functional dispersion correction (dft-d) for the 94 elements h-pu, J. Chem. Phys. **132**, 154104 (2010).

[2] T. Risthaus and S. Grimme, Benchmarking of london dispersion-accounting density functional theory methods on very large molecular complexes, J. Chem. Theory Comput. **9**, 1580 (2013).

[3] A. Simon, H. Borrmann, and J. Horakh, On the polymorphism of white phosphorus, Chem. Ber-Recl. **130**, 1235 (1997).

[4] H. Okudera, R. E. Dinnebier, and A. Simon, The crystal structure of γ-p4, a low temperature modification of white phosphorus, Z. Kristallogr. Cryst. Mater. **220**, 259 (2005).

Table II. Benchmark of different dispersion correction schemes for DFT-PBE0/TZVPP. a, b and c are given in Åunits, α, β and γ in degrees, and volumes V in $Å^3$. The percentage values in parentheses show the differences compared to experimental values. 90° angles are omitted.

Allotrope	Method	Disp. Corr.	atoms/cell	a (Å)	Err. (%)	b (Å)	Err. (%)	c (Å)	Err. (%)	b/a (arb. un.)	Err. (%)	c/a (arb. un.)	Err. (%)	c/b (arb. un.)	Err. (%)	α (deg.)	Err. (%)	β (deg.)	Err. (%)	γ (deg.)	Err. (%)	V ($Å^3$)	Err. (%)
White β	PBE0	-	24	5.77	5.3	11.43	5.9	11.72	6.9	1.98	-1.0	2.03	1.5	1.03	0.9	92.1	-2.4	99.8	0.2	101.9	1.3	743.3	19.2
		ZD		5.48	0.0	10.86	0.6	11.08	1.1	1.98	-0.9	2.02	1.0	1.02	0.4	93.9	-0.4	99.7	0.0	101.2	0.5	634.4	1.7
		ZD+ABC		5.51	0.5	10.91	1.1	11.12	1.4	1.98	-1.0	2.02	1.0	1.02	0.4	93.8	-0.5	99.8	0.2	101.2	0.5	641.6	2.9
	Exp. [3]			5.48		10.79		10.96		2.00		2.00		1.02		94.3		99.7		100.7		623.8	
White γ	PBE0	-	8	9.97	8.7	8.93	7.1	5.5	2.2	0.90	-1.5	0.56	-6.0	0.62	-4.6	-	-	89.7	-0.7	-	-	493.7	18.8
		ZD		9.25	0.9	8.25	-1.1	5.45	0.4	0.89	-2.0	0.59	-0.5	0.66	1.6	-	-	90.5	0.2	-	-	415.8	0.1
		ZD+ABC		9.30	1.4	8.25	-1.1	5.45	0.4	0.89	-2.0	0.59	-0.5	0.66	1.4	-	-	90.5	0.2	-	-	421.3	1.4
	Exp. [4]			9.17		8.34		5.43		0.91		0.59		0.65		-		90.3		-		415.5	
Red fibrous	PBE0	-	42	12.95	6.1	13.02	0.2	7.62	7.7	1.01	-5.6	0.59	1.5	0.59	7.5	113.8	-2.8	109.1	2.7	97.7	-0.2	1057.5	16.1
		ZD		12.27	0.6	13.00	0.1	7.11	0.5	1.06	-0.5	0.58	-0.1	0.55	0.4	116.9	-0.1	106.4	0.1	97.9	0.0	922.8	1.3
		ZD+ABC		12.36	1.3	13.01	0.1	7.15	1.0	1.05	-1.1	0.58	-0.3	0.55	0.9	116.6	-0.3	106.8	0.4	97.9	-0.1	936.1	2.8
	Exp. [5]			12.20		12.99		7.08		1.06		0.58		0.55		117.0		106.3		97.9		911.0	
Hittorf's Violet	PBE0	-	84	9.27	0.7	9.23	0.9	23.89	5.7	1.00	0.2	2.58	5.0	2.59	4.8	-	-	104.8	-1.2	-	-	1976.6	8.0
		ZD		9.22	0.1	9.13	-0.2	22.65	0.2	0.99	-0.3	2.46	0.1	2.48	0.4	-	-	106.0	-0.1	-	-	1832.9	0.2
		ZD+ABC		9.23	0.2	9.15	0.0	22.78	0.8	0.99	-0.3	2.47	0.6	2.49	0.8	-	-	105.8	-0.3	-	-	1850.6	1.1
	Exp. [6]			9.21		9.15		22.60		0.99		2.45		2.47		-		106.1		-		1829.8	
Black	PBE0	-	4	3.30	-0.2	10.97	4.8	4.50	2.8	3.32	4.9	1.36	3.0	0.41	-1.9	-	-	-	-	-	-	163.2	7.4
		ZD		3.30	0.1	10.61	1.3	4.42	0.9	3.21	1.5	1.34	1.1	0.42	-0.4	-	-	-	-	-	-	154.8	1.9
		ZD+ABC		3.31	-0.1	10.66	1.8	4.44	1.3	3.22	1.9	1.34	1.4	0.42	-0.5	-	-	-	-	-	-	156.3	2.9
	Exp. [7]			3.31		10.47		4.38		3.16		1.32		0.42		-		-		-		151.9	

[a] A

[b] B

[5] M. Ruck, D. Hoppe, B. Wahl, P. Simon, Y. Wang, and G. Seifert, Fibrous red phosphorus, Angew. Chem. Int. Ed. **44**, 7616 (2005).

[6] H. Thurn and H. Krebs, Über struktur und eigenschaften der halbmetalle. xxii. die kristallstruktur des hittorfschen phosphors, Acta Crystallogr. B **25**, 125 (1969).

[7] L. Cartz, S. R. Srinivasa, R. J. Riedner, J. D. Jorgensen, and T. G. Worlton, Effect of pressure on bonding in black phosphorus, J. Chem. Phys. **71**, 1718 (1979).
